# Women’s Empowerment in Iran: A Review Based on the Related Legislations

**DOI:** 10.5539/gjhs.v6n4p226

**Published:** 2014-04-20

**Authors:** Roksana Janghorban, Ali Taghipour, Robab Latifnejad Roudsari, Mahmoud Abbasi

**Affiliations:** 1 Student Research Committee, Department of Midwifery, School of Nursing and Midwifery, Mashhad University of Medical Sciences, Mashhad, Iran; 2 Health Sciences Research Center, Department of Biostatistics and Epidemiology, School of Health, Mashhad University of Medical Sciences, Mashhad, Iran; 3 Evidence-Based Care Research Centre, Department of Midwifery, School of Nursing and Midwifery, Mashhad University of Medical Sciences, Mashhad, Iran; 4 Medical Ethics and Law Research Center, Shahid Behshti University of Medical Sciences, Tehran, Iran

**Keywords:** legislation, women, empowerment, Iran

## Abstract

Women’s empowerment can be defined as a change in the circumstances of a woman’s life, which enables her to raise her capacity to manage more enriched and rewarding life. Improvement in women’s empowerment is a salient issue to achieve the Millennium Development Goals. National laws are influential factors in promoting women’s empowerment. Lack of awareness of legal and constitutional provisions and failure to recognize it, is a factor that hinders the process of empowerment. This paper provides a review based on Iranian legislations which have considered various aspects of women’s empowerment. Although this work has specifically dealt with women’s needs, it encompasses a right–based approach to women’s empowerment suggested by the United Nations Fund for Population Activities. However, there is still a great need for further inquiries in the area of legislations concerning women’s empowerment around the world in general and Iran in particular.

## 1. Introduction

Empowerment is an active and multidimensional process which enables women to realize their full identity and powers in all spheres of life (Pillai, 1995). There are five issues relating to women’s empowerment: a woman’s sense of self-value and respect; the right to have choices and decision making power; access to various opportunities, prospects and means; the right to have the power to control their own lives; and the ability to influence trends in social change to create more social and economic orders, both nationally and internationally (Secretariat of the United Nations, 2001). The Program of Action, International Conference on Population and Development (PoA, ICPD) stresses that the empowerment of women and the expansion of their political, social, economic, and health status are not only significantly important issues for themselves, but are vital for the achievement of sustainable human development (United Nations Population Information Network, 1994c). The most impressive element for the development is human talent of a country. Women make up one-half of the potential talents in the world. Therefore, national development greatly depends on how the society provides opportunities to flourish its females’ talents and then, gets advantage of their skills and productivity (Jager & Rohwer, 2009). In recent years, the empowerment of women has been recognized as the most fundamental concern in determining women’s status and position. Therefore, the women’s empowerment is a critical aspect of promoting gender equality (United Nations Population Fund [UNFPA], 2011). It is fulfilled when the individuals’ rights, responsibilities, and opportunities are not influenced by the gender- based judgment of being born male or female (Jager & Rohwer, 2009).

In the Iranian Constitution like many other countries, the principles of women’s empowerment and gender equality have been preserved. The Constitution of the Islamic Republic of Iran (The rights of the people), Article 20 states that under Iranian law, all citizens of the country, both men and women, are equally protected against the law and have all the political, economic, social, and cultural rights in accordance with Islamic rules (Article 20, 1928). It is notable that apart from the fundamental rights guaranteed in the constitution of the Islamic Republic of Iran for all citizens in 1928, women’s rights have clearly been spelt out in the nation’s constitution. In the preamble of the Iranian constitution, in the section of ‘Woman in the Constitution’, it has been indicated that:

Through the creation of Islamic social infrastructures, all the elements of humanity that served the multifaceted foreign exploitation shall regain their true identity and human rights. As part of this process, it is only natural that women should benefit from a particularly large augmentation of their rights because of the greater oppression from which they suffered under the old regime (Woman in the Constitution, 1979).

The constitution Article 21 clearly states that the government must ensure the rights of women in all spheres and is responsible for creating favorable contexts for the growth of women’s personalities and the revitalization of their material and moral rights (Article 21, 1928). The Iranian Constitution has also drawn special attention to the role of women in the family. In this regard it states that: ‘The family is the fundamental unit of society, and the main center for the growth and edification of human beings…’. This view of the family unit safeguard women from being considered as an object or instrument in the service of promoting consumerism and exploitation. Not only does a woman recover, thereby, she also assumes a pioneering social role, becoming a fellow partner alongside a man in all crucial aspects of life. As a result of the great responsibilities that a woman takes upon herself, she is accorded great value and nobility in Islam (‘Woman in the Constitution’, 1979). The primary object of these legislative principles is to bring about the advancement, development, and empowerment of women in Iranian society. The present paper attempts to provide a review based on Iranian legislations which have considered various aspects of women’s empowerment including access to education, financial autonomy, sexual and reproductive rights, and political participation.

## 2. Educational Empowerment

Education, which means having the minimum ability to read and write, provides skills which enable people to earn a living, as well as the circumstances in which the ability to think for one’s self, communicate and experience life more completely is valued (Gallaway & Bernasek, 2004). United Nations Population Information Network has emphasized: ‘Education is one of the most important means of empowering women with the knowledge, skills and self-confidence necessary to participate fully in the development process’ (United Nations Population Information Network, 1994b). One of the 2000 United Nations Millennium Development Goals (MDGs) is also to achieve primary education for every child by 2015. Another MDGs Goal, i.e., ‘To promote gender equality and empowering women’, centers on eliminating inequality between genders in primary and secondary education (United Nations [UN], 2000). Policies focusing on primary education for girls are certainly the best way to increase literacy among females. A complement to this policy is the provision of literacy training in women in order to improve their labor market outcomes in the short run and to contribute to their empowerment and the empowerment of their daughters in the long term. These targets have been considered in existing laws in Iran as well.

The Constitution of the Islamic Republic of Iran (general principles) Article 3, has a special focus on free education and training for everyone at all levels as well as facilitation and expansion of higher education (Article 3, 1928). The law on the Socio-cultural Council of Women, Article 1, has focused on elevating the level of public knowledge and literacy and also on adopting the appropriate policies in the fields of women’s K-12 and Higher Education (Article 1, 1997). In order to cancel the restrictions of girls admission in certain university courses, it is usually permissible for female volunteers to study in any field they choose unless they are faced with practical prohibition (Decree on Cancellation, 1989). Policies of the Islamic Republic of Iran on Women’s Employment, Article 7 have emphasized the provision of facilities required for utilizing the capabilities of educated women as well as expert and specialist women (Article 7, 1992). Article 11 of the same document emphasizes that technical and vocational education and appropriate occupational opportunities shall be facilitated for women who are the breadwinners of their families (Article 11, 1992).

## 3. Economic Empowerment

Economic empowerment implies that women are able to take on productive activities that confer some degree of financial independence, however, small and burdensome they may be initially (United Nations Educational, Scientific and Cultural Organization [UNESCO], 1993). ICPD, PoA, 1994, has highlighted: ‘As women are generally the poorest of the poor … eliminating social, cultural, political and economic discrimination against women is a prerequisite of eradicating poverty … in the context of sustainable development’ (United Nations Population Information Network, 1994a). In many societies around the world, women never entirely belong to themselves; they own less than one percent of the world’s property while they compose 66 percent of the global workforce and produce half of the food (UN Women, 2009; United Nations Development Programme [UNDP], 2011). In the circumstances where women have no control over money, they are unlikely to provide health care for themselves or their children (CARE, 2005).

To foster women’s economic empowerment, the government of Iran has worked to set laws to support their livelihoods, financial provisions in divorce cases, and women’s access to labor opportunities (Article 1107, 1928; Article 1082, 1928; Article 1092, 1928; Article 7, 1998; Article 1118, 1928; Article 1106, 1928; Article 1111, 1928). As soon as the marriage is contracted, the wife becomes the possessor of the marriage portion (*Mahriyya*)^Note 1^ and can do anything with it, as she wishes (Article 1082, 1928), and if the husband divorces his wife before intercourse, the wife will be entitled to receive half of the marriage portion (Article 1092, 1928). If the marriage portion was in Rial (the Iranian currency) at the time of marriage, it is calculated and paid, whenever the wife requests it, based on changes in the annual price index, announced by the Central Bank of the Islamic Republic of Iran (Article 1082, 1928). If the husband appeals to the court to divorce his wife, the court is responsible to determine how the marriage portion will be paid (Article 7, 1998). The wife can independently possess and do anything with her own property (Article 1118, 1928). In a permanent marriage the wife’s alimony *(Nafaqa)* must be paid by the husband (Article 1106, 1928). Alimony is the money which covers all ordinary requirements of the wife including housing, clothing, food, house furniture, and appliances appropriate to the wife’s social status; and also money for employing a domestic servant if she is accustomed to having one or needs one due to being ill or disabled (Article 1107, 1928). In the case of the husband’s refusal to pay alimony, the wife can appeal to a court of law in which the judge will determine the amount of the alimony and order the husband to pay it (Article 1111, 1928). In addition, various laws have been legislated for the economic empowerment of women in the social framework (Article 2, 1992; Article 3, 1992; Article 4, 1992; Article 9, 1992). Since women’s employment in cultural, social, economic and administrative occupations is a prerequisite for the achievement of social justice and the advancement of a society, special attention must be paid to this issue (Article 2, 1992). For better management of all household affairs and fulfillment of social responsibilities, cooperation of all family members with each other is of special significance (Article 3, 1992). The circumstances of the women’s workplace in society must be prepared in a manner in which the grounds for women’s spiritual, scientific, and professional development are provided. Furthermore, women’s faith, personality, dignity as well as mental, spiritual, and physical health should not be damaged in any way (Article 4, 1992). Also, in an equal condition the work of men and women should be valued equally and equal salary and benefits must be regarded as well (Article 9, 1992). Considering the central role that the state of the Islamic Republic of Iran gives to the stability of the family as well as women in training and procreation inside the home, the required regulations and facilities proportionate to ‘motherly occupation’ including: paid maternity leave, reduction in working hours, retirement benefits with less length of service, job security, and social security during unemployment period, sickness, or inability to work have been ratified (Article 10, 1992).

## 4. Political Empowerment

According to UNESCO, ‘political empowerment would encompass the ability to organize and mobilize for a change. Consequently, an empowerment process must involve not only individual awareness but collective awareness and collective action. The notion of collective action is fundamental to the aim of attaining social transformation’ (UNESCO, 1993). Women’s political participation is an important issue in the context of empowerment. Desai and Thakkar (2001) have discussed women’s political participation, legal rights, and education as mechanisms for their empowerment (Desai & Thakkar, 2001). Based on conventional analysis, women’s political participation means activities related to electoral politics like voting, campaigning, holding party office, and competing in an election. But in a broader sense it encompasses all voluntary actions intended to influence the making of public policies, the administration of public affairs, and the choice of political leaders at all levels of government (Nayak & Mahanta, 2009).

Legislation in Iran has created a new political atmosphere in which women’s voices can be heard and a consensus can be forged. It has also endeavored to empower women by training them to negotiate on a global basis, particularly since the end of the Iraq war, which shaped a resurgence in women’s activity. The United Nations Children’s Fund (UNICEF) has remarked that numerous government organizations are functioning primarily in areas such as legislation and research as regards the legal and socio-economic status of women (United Nations Children’s Fund [UNICEF], 1993). According to Iranian women’s employment policies, government is committed to encourage educated and experienced specialist women to occupy managerial and staff positions, in order to utilize women’s effectiveness at high executive levels (Article 6, 1992). Currently, in Iran eight women are working as Members of Parliament, which is promising in terms of taking into account the issue of political empowerment of women in Iran. This demonstrates the potential of Iranian women in participating in decision making processes at the highest policy level.

## 5. Reproductive and Sexual Empowerment

Women’s empowerment is an essential requirement for sound reproductive health (Secretariat of the United Nations, 2001). The concept of Sexual and Reproductive Health and Rights (SRHR) was first declared as a human right at ICPD, 1994. The definition of reproductive health that was adopted also embodied sexual health (World Health Organization [WHO], 2004).

According to the World Health Organization (WHO), sexual health has been defined as: ‘a state of physical, emotional, mental, and social well-being related to sexuality; it is not merely the absence of disease, dysfunction or infirmity’. Sexual health requires ‘a positive and respectful approach to sexuality and sexual relationships, as well as the possibility of having pleasurable and safe sexual experiences, free of coercion, discrimination, and violence’. To achieve and sustain sexual health, the sexual rights of all persons must be respected, protected, and fulfilled. Sexual rights are defined by WHO as: ‘human rights that are already recognized in national laws, international human right documents, and other consensus documents’. These include the right of all persons, without bias, inequity, and violent behavior, to the highest possible standard of health with respect to sexuality, including access to sexual and reproductive health care services; to request, receive and pass on information as regards sexuality; sexual education; respect for bodily integrity; choice of partner; to elect whether or not to be sexually active; to engage in sexual relations by mutual consent; marriage by consent; to choose whether or not to have children in addition to deciding the proper time and situation in which to do; and engage in a satisfying, secure, and gratifying sexual life (WHO, 2002).

In Iran, reproductive and sexual rights are well provided and laws and penalties are clearly spelt out for offenders. For instance, the following defects in a man are grounds for the right of marriage termination for the wife: 1) Impotence that continues within one year after the wife’s application to the judge, 2) If the husband’s genital organ is cut off, and 3) Castration (Article 1122, 1928). Also, madness and impotency of the man is a basis for the right of marriage cancellation for the wife, even if it happens after the marriage contract (Article 1125, 1928). If the husband is afflicted with a venereal disease after the marriage contract, the wife is entitled to refuse sexual intercourse with him and this refusal will not terminate her right to alimony (Article 1127, 1928). Additionally, according to the Iranian Family Protection law, no man is allowed to remarry without the permission of the court or his first wife. The courts only grant permission under specific circumstances such as serious and incurable illness of the first wife (Article 16, 1974).

Apart from the Iranian constitution, there are other laws that protect women against violence in Iran. Iranian Civil Law and Related Regulations (Physical Capacity for Marriage), Article 1041 affirms that girls marriage is forbidden before puberty without permission of a guardian (Article 1041, 1928). This means that if a man marries a pre-pubertal girl, against the regulations of Article 1041 of the Civil Code, he shall be sentenced to correctional imprisonment from six months to two years, and in a case where the girl has not yet completed 13 years of age, the person concerned shall be sentenced to correctional imprisonment of at least 2-3 years. If sexual activity causes permanent morbidity for the wife, the husband shall be sentenced to prison for 5-10 years with physical labor (Article 3, 1931). Iranian Civil Law and Related Regulations (The Mutual Rights and Duties of the Married Couple towards Each Other), Article 1115 declares that if the wife’s living with her husband in the same house is associated with fear of physical harm, financial loss or prestige damage for the wife, she can then choose a separate habitat, and in the case where suspicion of harm is proven, the court will not permit the wife to return to the husband’s house and as long as she is excused from returning, the husband is obliged to pay the alimony (Article 1115, 1928).

In addition, Iranian women are protected by law from Female Genital Mutilation (FGM), the harmful procedure that violates women’s human rights. Article 479 of the Islamic penal code declares: ‘if a woman’s genitalia is totally severed, she shall be entitled to her full blood money *(Ghesas)*^Note 2^ and if only half of her genitalia is severed, half of her blood money is due to her’ (Article 479, 1996).

There are also specific laws against trafficking of women and girls in Iran. Anti-trafficking law, Article 2 emphasizes that women’s trafficking, even with their consent, results in imprisonment from two to ten years and being sentenced to pay fines equal to double covered or alternatively property resulting from a felony (Article 2, 2004). Pornography is also illegal in the Islamic Republic of Iran. For this reason, the new law of the computer crime legislation has been approved in Iranian parliament on 19^th^ November 2008, which came into force on 29^th^ June 2009. According to this law, producing, publishing, and distributing of any real or unreal images, audio recordings or writings that show full nudity of women or men, their genitals or their engagement in a sexual act are crimes against public morality and chastity. The person involved in this offence may be punished by imprisonment of 91 days to two years or a fine of 5,000,000 to 40,000,000 Rial or both (Article 14, 2008).

One of the most debated themes in reproductive rights is abortion. Currently, Iranian law permits therapeutic abortion after a definite diagnosis by three experts and its confirmation is done by the Forensic Medicine Organization (FMO). This decision might be based on fetal diseases leading to suffering or illness for the mother due to disability such as fetus malformation or retardation, or based upon life-threatening maternal diseases. In the Therapeutic Abortion Act, The Islamic Republic of Iran Parliament declares that abortion may be performed before acquiring a human soul *(ensoulment*)^Note 3^ with the consent of the woman. In this case neither accountability, legal or otherwise, nor chastisement would be directed towards the physician (The Therapeutic Abortion Act., 2005). Offenders practicing contrary to the provisions of the Act will be punished by imprisonment from two to five years and the compensatory payment (*diyyeh*)^Note 4^ according to the penalties of Islamic law. The amount of payment is calculated according to the fetus stage of growth (Article 624, 1996).

Taking into consideration the substantial requests of infertile couples, the Iranian parliament approved a law entitled Embryo Donation to Infertile Spouses Act in 2003. Under the new law, the donation of an embryo is allowed under specified conditions (The Embryo Donation, 2003). The act has been quite effective in preventing the break-up of many marriages in recent years, though no data has as yet been published. The cost of Assisted Reproductive Technologies (ARTs) is high in general, although the costs in the Islamic Republic of Iran are, by far, much lower than many other countries. Due to this, the Supreme Council of Insurance has considered covering the cost of one cycle of ART treatment’ (Zahedi & Larijani, 2008), so it would be free of charge for infertile couples. In addition, Iranian legal system has accepted surrogacy as a method which could provide the possibility of parenthood for some infertile couples. Article 10 of the Iranian Civil Act about a surrogacy contract between a surrogate woman and intended parents declares: ‘Private contracts will be effective to those who conclude them if they are not in contrast with the law’ (Samavati Pirouz & Mehra, 2011; Article 10, 1928).

## 6. Discussion

Regardless of the many international agreements formally confirming women’s human rights, women are still much more likely than men to suffer from poverty and illiteracy, as well as having less access to medical care, property rights, credit, education, and employment. They are far less likely than men to be politically active and, to a greater extent, victims of domestic violence. One vital aspect of promoting equal opportunity between the sexes is the empowerment of women. It is vital to sustainable development and the realization of human rights for all (UNFPA, 2008; UNDP, 2007).

United Nations Fund for Population Activities (UNFPA) is committed to actions that attack poverty, illiteracy, and powerlessness; especially among women. ‘About half of the UNFPA program countries have developed strategies to provide women with economic opportunities. The Fund has established partnerships with parliamentarians in developing countries for political and legislative support for population and development challenges, of which the empowerment of women is central’ (UN Women, 2010).

In Iran, the trend of incorporating women’s affairs in economic, social, cultural, and political development plans shows an upward trend from the first development plan after the victory of the Islamic Revolution to the present time. The article 158 of the Third Development plan was the first initiative for the streamlining of women’s empowerment approaches in the major plans. The most important points of the Article 158 of the Third Development plan are: 1) Identification of the educational and sport needs of women on the basis of Islamic principles and promoting their role in the future development of the country, 2) Promoting women’s job opportunities, 3) Facilitating women’s access to legal and juridical affairs, and 4) Supporting, launching, and establishment of women’s non-governmental organizations with emphasis on support for the women who are heads of family as well as women who are lacking legal protection in the less developed regions of the country. Attempts have also been made to streamline and institutionalize women’s empowerment approaches throughout the Fourth Development plan (2005 – 2009), which is binding on all governmental organizations, and with the earnestness of the civil society institutions, are followed seriously (World Bank, 2009).

It is crucial that attention be paid to the goal of eliminating illiteracy among women in developing efforts. Equal access to education for women and girls has been guaranteed in the Iranian laws as a right for all its citizens (Article 3, 1928). Literacy rate for both sexes had an increasing trend over the years from 1991 to 2010 in Iran. According to the World Bank report, female adults’ literacy rate (ages 15 and older) in the country was 77 percent compared with 87 percent in the case of males in 2008 (World Bank, 2010, 2012). According to the global gender gap report 2012, educational attainment score of Iran was 0.953. In this score, the gender gap in current access to education has been captured through female-to-male ratio in primary, secondary, and tertiary education. Iran has showed no gap in primary and tertiary level (World Economic Forum, 2012). Progress has been made in pursuit of educating girls, yet more progress is needed to obtain said objective. However, in terms of enhancing life for women and children in the developing world, a better understanding of how women could have access to literacy training that creates significant benefits for them is vital. There are two-stages in this process. The first stage entails focusing on the link between literacy and prospective labor market outcomes relating to women, while the second requires research into successful programs and schemes and understanding how they can be applied in different situations (Gallaway & Bernasek, 2004).

Women’s wage is important for economic growth and the well-being of families. But women are often hindered by obstacles such as limited availability ‘to education and vocational training, heavy workloads at home, unpaid domestic and market activities, and labor market discrimination’. These obstacles hinder women, forcing them to limit their participation in paid economic activities, and even when women work, cause them to be less productive and accept lower pay. When women are in paid employment, they tend to be concentrated in the nonagricultural sector (World Bank, 2010, 2012; Chen et al., 2005). In Iran, 16 percent of women work in wage employment in the nonagricultural sector. However, in many developing countries women make up a large proportion of agricultural employees, often as unpaid family workers. Female employment in the country in the agricultural sector is 33 percent versus 21 percent of males. Among people who work without wages, it is more likely that women rather than men end up as unpaid family workers, while men are more likely to be self-employed or employers in comparison to women. This pattern exists in Iran because 29.7 percent of women and 4.8 percent of men are unpaid family workers (World Bank, 2010, 2012). There are several reasons for this issue. Firstly, few women have access to credit markets, capital, and land, which may be required to start a business. Secondly, cultural norms may discourage women from having the confidence or possibility of working for themselves or supervising other workers. Also, women may also be hindered by time constraints as a result of their traditional family responsibilities (World Bank, 2010, 2012; Saigol, 2011). On the economic participation and opportunity, Iran has closed nearly 41 percent of its economic gender gap. In 2012, World Economic Forum reported the sub index scores of Iranian women’s economic participation as such: labour force participation: 0.44, wage equality for similar work: 0.63, women’s share of legislators, senior officials, and managers: 0.15, and professional and technical workers: 0.50 (World Economic Forum, 2012).

In relation to political empowerment, the proportion of parliamentary seats held by women has increased steadily since the 1990s. The most impressive gains have come in Latin America and the Caribbean, the Middle East and North Africa and South Asia, where women’s representation rose 30–50 percent over 1990–2009. Although countries in the Middle East and North Africa have made considerable improvements and advances in the political sector, women continue to hold less than 10 percent of parliamentary seats, the lowest among all regions (World Bank, 2010, 2012). Presently, approximately 31 women have become heads of state or government in the world (Worldwide guide, 2013). It seems that many factors are responsible and decisive in the election of women candidates such as literacy, financial status, liberal family atmosphere, support of other members of the family, and strong personality. Since most of the women lack access to the aforementioned issues, few women are able to get on tickets, and even fewer get elected from this handful of women candidates. In Iran, women gained right to vote in January 1963 (Shahidian, 2002). The Iranian women’s political participation has increased since the 8^th^ parliament. The trend shows the political presence of women as the Ministry of Health, Vice president of the Islamic Republic of Iran for Legal Affairs and Science and Technology, Head of the Environment Protection Organization, and Head of the Center for Women’s Participation (CWP) (Shojaei, Samsu & Asayeseh, 2010). Women hold three percent of parliamentary seats in the 10^th^ Islamic Consultative Assembly *(Majlis)*. Nevertheless, no Iranian women have ever held an elected executive office (prime minister or president) for the last 50 years (World Economic Forum, 2012).

In terms of sexual and reproductive empowerment, the global burden of ill-health due to reproductive and sexual problems amounts to almost 20 percent in women and 14 percent in men. This data, indicative of the high proportion of ill-health, illustrates the gravity of sexual and reproductive health as a serious public health problem (Ekdahl, 2009). Sex ratio at birth and female-to-male healthy life expectancy ratio are two significant variables that display gender gap in health and survival (World Economic Forum, 2012). The unusually high sex ratio (number of males divided by the number of females) could show the prevalence of “missing women” in countries where sex-selection approach is a serious issue (Klasen and Wink, 2003). In 2012, the overall population sex ratio (female/male) was 0.95 in Iran. In the same year, the ratio of female-to-male healthy life expectancy was reported 1.03 (World Economic Forum, 2012). It provided an estimation of years that Iranian women could expect to live in ‘full health’, excluding the years lived at less than full health due to disease, violence, and malnutrition (Salomon, Mathers & Murray, 2001). In 2012, the World Bank reported some Iranian reproductive health indicators as such: Total fertility rate: 1.7 births per woman; contraceptive prevalence rate: 79 percent; births attended by skilled health staff: 97 percent; maternal mortality ratio: 30 per 100000 live births (World Bank, 2010, 2012); mean age at marriage for women: 24 years; early marriage (aged 15-19): 17 percent, adolescent fertility rate: 31 births per 1000 girls aged 15-19, and female HIV prevalence (aged 15-49): 0.10 percent (World Economic Forum, 2012). Iran has tried to implement some programs towards ICPD targets for promoting women’s sexual and reproductive health (Roudi-Fahimi, 2002; Simbar, 2012).

## 7. Conclusion

In conclusion, law is a powerful instrument to advance the purpose of women’s empowerment; one strategy for formulating laws on women’s empowerment is to conceptualize issues into a separate national law. There are a number of national laws on women’s empowerment matters in Iranian jurisdictions, and many commentators believe that this approach can better clarify women’s empowerment issues within the legal framework of the State. Despite the enactment of numerous laws, women’s empowerment in some fields such as elected politicians needs more attention. With advancing conditions of equity between the sexes promoted by the national constitution, some planning and executive processes affecting women’s empowerment require review in order to assess their particular impact on women. Therefore, a realistic assessment of how the laws operate in practice will need to determine obstacles in women’s empowerment. Although good steps have been taken towards preserving empowerment in the spheres of education, economics, policy and reproductive and sexual rights issues in Iran since the Islamic Revolution.

## Notes

**Notes**

Note 1. Under Islamic c Law (shari‘a– Jurisprudence), at the time that marriage is performed, an amount of money/gold or property is specified and reflected on the marriage certificate as *Mahriyya*. This is usually a substantive amount, which serves as a financial backing/support for the woman particularly in case of divorce.

Note 2. *Ghesas* is a retaliated punishment in Islamic law in some crimes like murder penalty or body damage.

Note 3. *Ensoulment* is an Islamic concept, occuring at four months of pregnancy, when the spirit causes the emergence of potentiality for rational thought.

Note 4. According to Islamic penal code, *diyyeh* is the financial compensation a person must pay when he or she inadvertently causes physical injury to another.
